# Ischiofemoral impingement secondary to valgus intertrochanteric osteotomy: a case report

**DOI:** 10.1590/0100-3984.2013.0026

**Published:** 2017

**Authors:** Alice Duarte de Carvalho, Flávio Luís Garcia, Marcello Henrique Nogueira-Barbosa

**Affiliations:** 1 Fellow in Musculoskeletal Radiology, Radiology Division, Department of Clinical Medicine, Ribeirão Preto Medical School, University of São Paulo (FMRP-USP), Ribeirão Preto, SP, Brazil.; 2 Associate Professor, Department of Biomechanics, Medicine, and Rehabilitation of the Locomotor System, Ribeirão Preto Medical School, University of São Paulo, Ribeirão Preto, SP, Brazil.; 3 Associate Professor, Division of Radiology, Department of Clinical Medicine, Ribeirão Preto Medical School, University of São Paulo (FMRP-USP), Ribeirão Preto, SP, Brazil.

**Keywords:** Femoroacetabular impingement, Ischium, Femur, Osteotomy, Hip, Impacto femoroacetabular, Ísquio, Fêmur, Osteotomia, Quadril

## Abstract

We report an unusual case of ischiofemoral impingement secondary to valgus intertrochanteric osteotomy. The osteotomy was performed for treatment of epiphysiolysis of the left femoral head.

## Abstract (pt)

Nós relatamos um caso incomum de impacto isquiofemoral secundário a deformidade resultante de osteotomia intertrocantérica valgizante para tratamento de epifisiólise da cabeça do fêmur esquerdo.

## INTRODUCTION

The evaluation of the musculoskeletal system by imaging methods has been the object of a series of recent studies in the radiology literature of Brazil^(1-8)^. Various bone impingement syndromes are currently recognized as possible causes of hip pain, the most common being femoroacetabular impingement^(9-11)^. Ischiofemoral impingement is rare and involves impingement between the ischium and the lesser trochanter of the femur^(12)^. Initially, this phenomenon was described in three patients who had previously undergone hip surgery, of whom two had undergone hip arthroplasty and one had undergone valgus osteotomy of the proximal femur^(13)^. Ischiofemoral impingement also occurs in patients who have no history of hip surgery^(14,15)^. Other potential causes of this abnormal relationship include anatomical variability of the proximal femur, valgus hip^(9,13,14)^, prominent lesser trochanter, and a sessile osteochondroma^(14)^.

The objective of this report was to illustrate a rare case of ischiofemoral impingement. In the case reported, the condition developed chronically following valgus osteotomy to treat epiphysiolysis of the left femoral head.

## CASE REPORT

A 13-year-old female patient underwent valgus intertrochanteric osteotomy due to a history of epiphysiolysis of the left femoral head. At the age of 42, she underwent a physical examination, which showed her left leg to be approximately 1.5 cm shorter than her right. That same year, after imaging tests, she was diagnosed with osteoarthritis of the left hip associated with ischiofemoral impingement. At 45 years of age, the patient underwent total left hip arthroplasty, which resulted in significant improvement of the symptoms.

**Figure 1:**
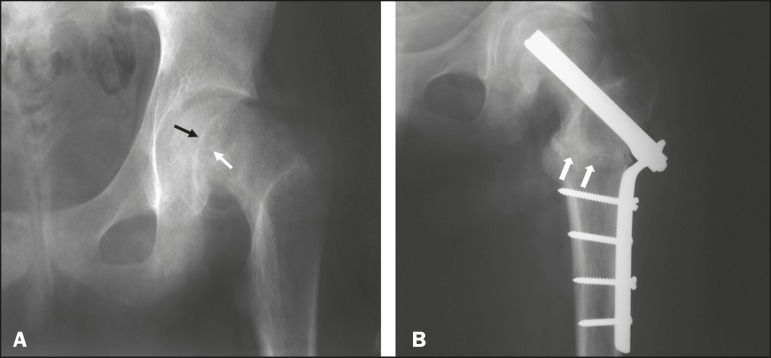
A: Initial radiograph of the left hip, in an anteroposterior view, showing signs of epiphysiolysis, with inferomedial displacement of the femoral head and an open physis (arrows). B: Radiograph of the left hip, in an anteroposterior view, after valgus intertrochanteric osteotomy; note the positioning of the osteotomy (arrows), fixed with plates and screws.

**Figure 2:**
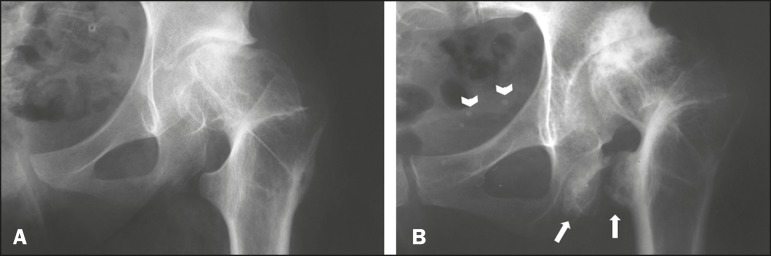
A: Radiograph of the left hip, in an anteroposterior view, approximately one year after valgus intertrochanteric osteotomy, showing signs of early hip osteoarthritis and a reduction in the distance between the lesser trochanter of the femur and the ischial tuberosity. B: Radiograph of the left hip, in an anteroposterior view, approximately 30 years after the initial procedure, showing exacerbation of the previous findings, with progression of hip osteoarthritis and narrowing of the ischiofemoral space (arrows).

The initial radiographs of the left hip showed femoral epiphysis, and the deformity was later corrected by the valgus intertrochanteric osteotomy (Figure 1). Images obtained approximately 30 years after the surgical intervention have shown evidence of advanced osteoarthritis of the hip. At that time, X-rays showed signs suggestive of ischiofemoral impingement with osteosclerosis and irregularity of the contours on the surfaces of the ischium and the lesser trochanter of the femur were also identified, together with a reduction of the space between the two bone structures (Figure 2B). The ischiofemoral space measured approximately 0.8 cm.

**Figure 3:**
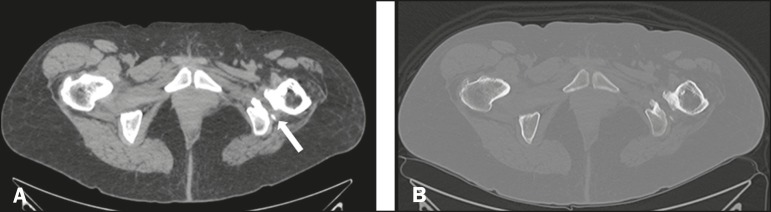
A: Computed tomography slice in the axial plane, with a soft tissue window, showing a reduction in the distance between the lesser trochanter and the left ischium, with signs of reactive hyperostosis and volume loss with fat replacement in the quadratus femoris muscle (arrow). B: Same slice shown in A, although with a bone window.

Computed tomography scans confirmed the presence of osteosclerosis and reactive bone proliferations in the ischium and the lesser trochanter of the femur, accompanied by a reduction in the quadratus femoris muscle space (Figure 3).

**Figure 4:**
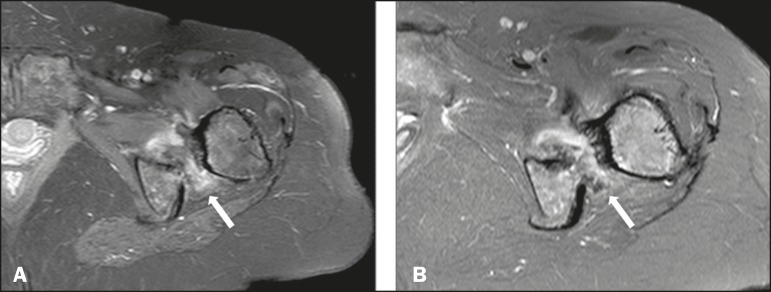
A: Contrast-enhanced T1-weighted magnetic resonance imaging scan with fat suppression, showing narrowing of the ischiofemoral space and enhancement of the soft tissues in the quadratus femoris muscle region (arrow). B: Axial T2-weighted sequence with fat suppression, showing a pattern of soft-tissue edema interposed between the minor trochanter and the ischial tuberosity (arrow).

Magnetic resonance imaging showed volumetric reduction and alteration of the signal of the quadratus femoris muscle, with discontinuity of its fibers and a pattern of edema in the T2-weighted images, those being signs of rupture of the muscle due to the ischiofemoral impingement (Figure 4).

## DISCUSSION

The diagnosis of ischiofemoral impingement is typically based on imaging findings, such as reactive, mirror-image bone changes in the ischium and the lesser trochanter of the femur, associated with reduction of the ischiofemoral space and partial or complete ruptures of the quadratus femoris muscle^(13-15)^. The hamstrings and iliopsoas tendons can also be involved^(14)^. In some patients with ischiofemoral impingement, a structure similar to the bursa or edema around the iliopsoas tendon can be seen^(15)^. Some patients also present muscle hypotrophy and fatty infiltration of the quadratus femoris muscle^(15)^.

In the literature, there is some controversy about whether or not quadratus femoris muscle lesions are associated with ischiofemoral impingement^(16,17)^. Torriani et al.^(15)^ demonstrated that the ischiofemoral space is significantly narrower in patients with quadratus femoris muscle abnormalities than in control subjects. The authors suggested that isolated alterations of that muscle can be multifactorial, ischiofemoral impingement representing one of the likely causes. Differential diagnoses, such as distension or rupture of the quadratus femoris muscle or late-onset exercise-induced muscle pain, can occasionally be excluded through clinical history-taking. The edema caused by rupture or distension most often occurs at the myotendinous junction, whereas the muscle edema caused by ischiofemoral impingement is diffuse and affects the ventral portion of the muscle^(12)^.

It should be borne in mind that the dimensions of the ischiofemoral space depend on the degree of rotation of the femur during image acquisition. To our knowledge, there have been no prospective studies of ischiofemoral impingement, and it is therefore possible that there are variations among the existing studies in terms of the positioning of the patients during imaging studies. With the hip in discrete adduction, extended, and with external rotation, the distance between the lesser trochanter and the ischium is usually 2.0 cm^(13)^. In the case presented here, that distance was approximately 0.8 cm. When the narrowing of the ischiofemoral space is congenital, it is usually bilateral and can be an asymptomatic incidental finding in some cases^(18)^.

In the study of ischiofemoral impingement conducted by Torriani et al.^(15)^, all of the patients were female, suggesting a possible relationship between ischiofemoral impingement and the anatomy of the female pelvis. In addition to having greater width and depth, the female pelvis is characterized by a greater distance between the ischial tuberosities^(15)^. Bilateral involvement is reported in approximately one third of patients with ischiofemoral impingement. The affected patients are typically older than are those with other types of hip impingement^(15)^.

To our knowledge, there have been no studies providing a detailed description of the clinical history and physical examination findings that characterize ischiofemoral impingement, although there have been reports that some patients with ischiofemoral impingement present with pain in the groin and medial thigh, induced by external rotation of the hip, in adduction or in extension^(13)^. Patients can also present tenderness and pain upon palpation of the lesser trochanter region^(13)^. The pain can radiate distally because of irritation of the adjacent sciatic nerve^(13-15)^.

Complete pain relief can be achieved after resection of the lesser trochanter^(13)^, supporting the hypothesis that abnormal contact between the trochanter and the ischium is responsible for the symptoms of ischiofemoral impingement. Although some authors recommend surgical decompression of the quadratus femoris muscle with minor trochanter resection, others favor conservative treatment or computed tomography-guided steroid injection^(12)^.

In the case described here, the femoral valgus related to the previous corrective osteotomy was associated with the subsequent development of ischiofemoral impingement. Ischiofemoral impingement secondary to hip arthroplasty has previously been reported^(13)^.

As previously stated, ischiofemoral impingement is a rare condition. Nevertheless, we believe that it should be considered in the differential diagnosis of pain after hip surgeries.
